# Supplementary material for: Probing nanoscale oxygen ion motion in memristive systems
Source: Nat Commun. 2017 May 4;8:15173. doi: 10.1038/ncomms15173 (PMC5418605; doi:10.1038/ncomms15173)
Supplement: Supplementary Information — Supplementary Figures, Supplementary Notes and Supplementary References [file ncomms15173-s1.pdf]

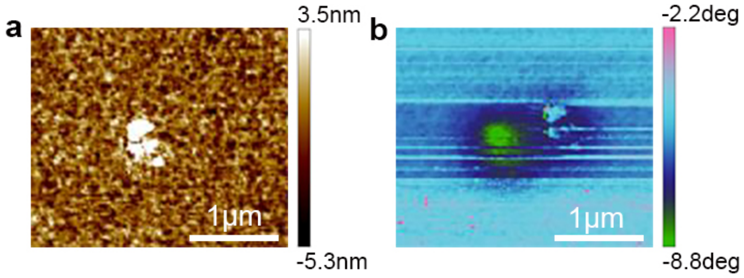

Supplementary Figure 1 | Phase detection EFM measurements verifying the nature of the accumulated charges. (**a**) Topographic profile and (**b**) EFM phase image of the region after being stimulated by a positive voltage sweeping up to 9 V. Negative phase shift was observed in the electrically stimulated region by a positive voltage, indicating attractive force between the probe and the sample.

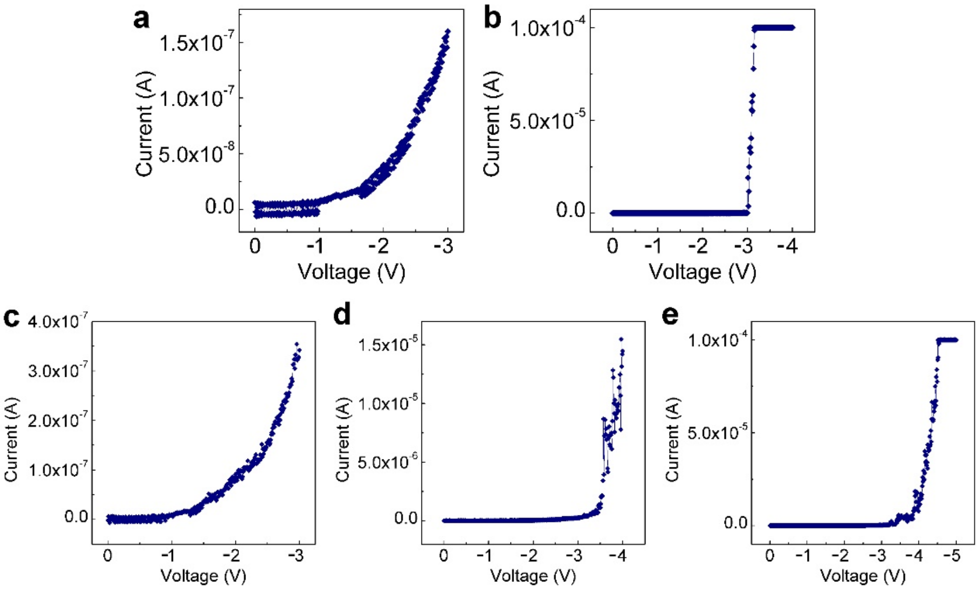

Supplementary Figure 2 | *I*–*V* characteristics of the HfO_2/TiN sample during negative voltage sweeps. (**a**–**b**) *I*–*V* characteristics during the voltage sweeping to –3 V (**a**) and –4 V (**b**) corresponding to the EFM measurements in Figs. 3a–c. (**c**–**e**) *I*–*V* characteristics during the voltage sweeping to –3 V (**c**), –4 V (**d**) and –5 V (**e**) corresponding to the EFM measurements in Figs. 3d–f.

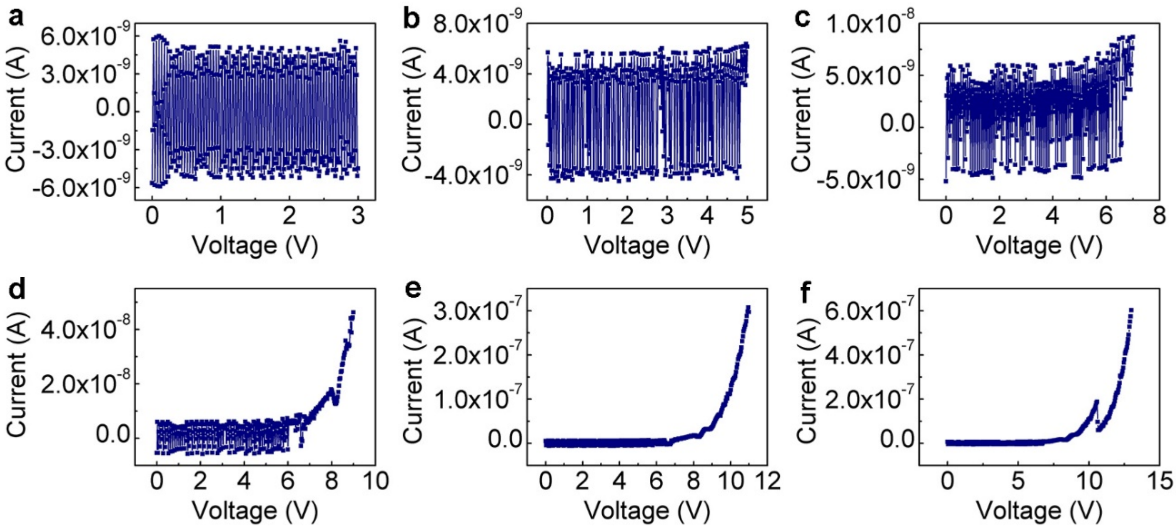

Supplementary Figure 3 | *I*–*V* characteristics of Al_2O_3/TiN samples under different programming conditions. *I*–*V* characteristics during the voltage sweeping to 3 V (**a**), 5 V (**b**), 7 V (**c**), 9 V (**d**), 11 V (**e**), and 13 V (**f**) corresponding to the topographic and EFM measurements shown in Fig. 4.

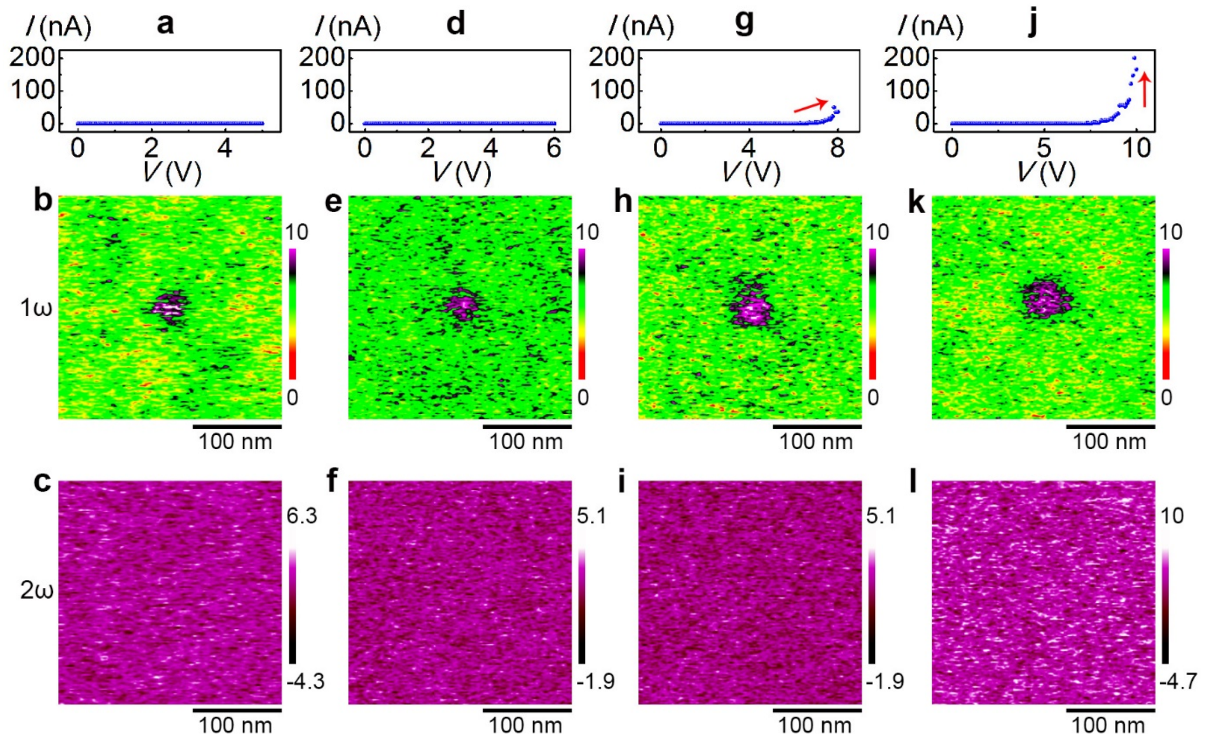

Supplementary Figure 4 | C-AFM and EFM measurements in ultrahigh vacuum condition. (**a–c**) Electrical (**a**), 1ω (**b**) and 2ω (**c**) measurements on the region stimulated by voltage sweeping up to 5 V during preceding C-AFM measurements. (**d–f**) Corresponding electrical and EFM results on the region stimulated by voltage sweeping up to 6 V during preceding C-AFM measurements. (**g–i**) Corresponding electrical and EFM results on the region stimulated by voltage sweeping up to 8 V during preceding C-AFM measurements. (**j–l**) Corresponding electrical and EFM results on the region stimulated by voltage sweeping up to 10 V during preceding C-AFM measurements.

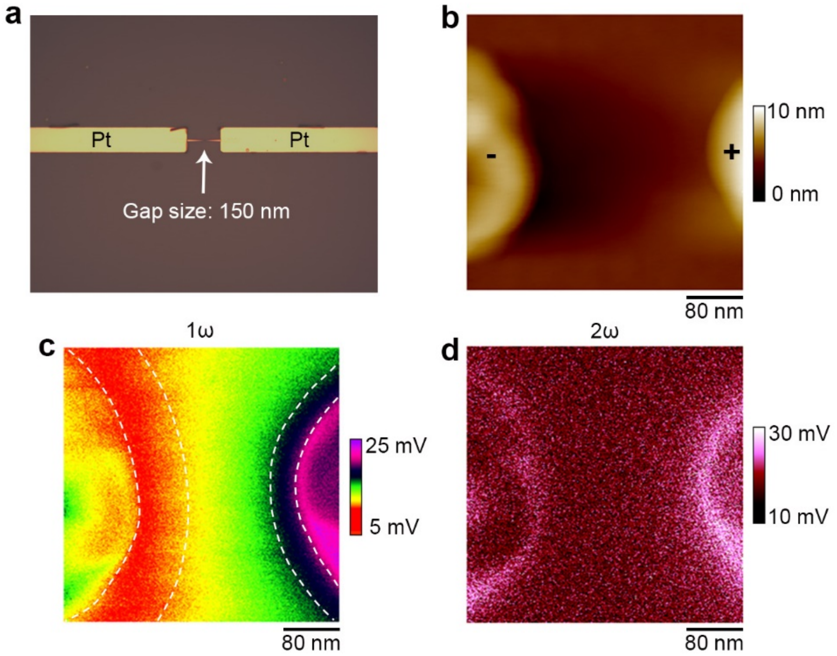

Supplementary Figure 5 | Electrostatic force microscopy on lateral devices. (**a**) Optical micrograph of the lateral device fabricated by e-beam lithography. The arrow indicates the device with a nominal gap size of 150 nm, as enlarged in (**b**). (**b**–**d**) Topographic (**b**), 1ω (**c**) and 2ω (**d**) measurements on the lateral device in (**a**) with a positive voltage sweep applied on the right electrode in preceding C-AFM measurement.

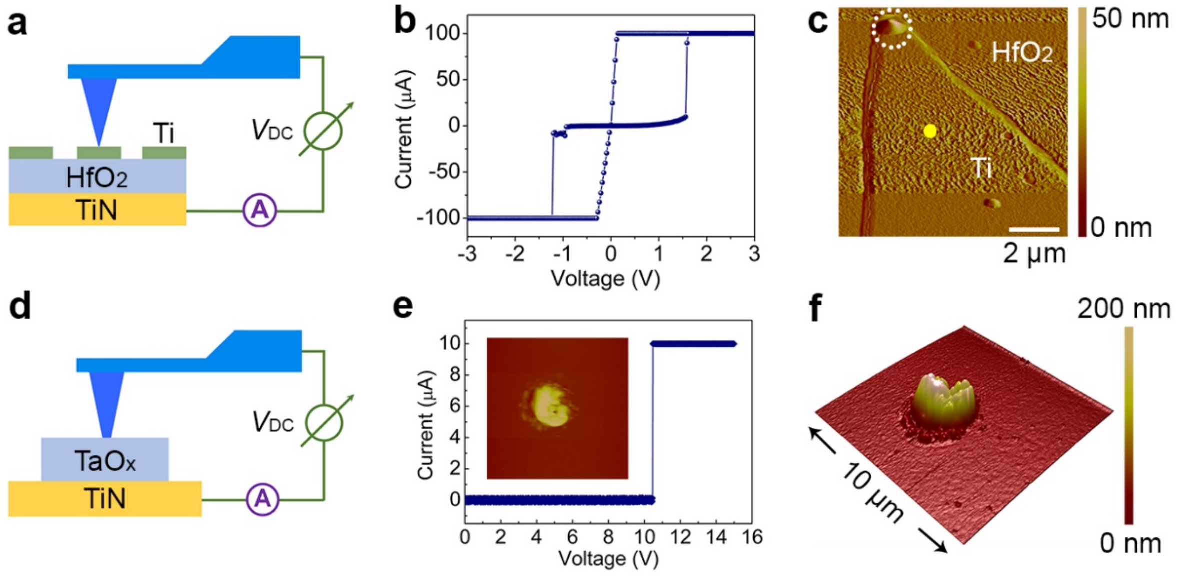

Supplementary Figure 6 | C-AFM measurements on Ti/HfO_2/TiN and TaO_x/TiN structures, showing oxygen gas eruption and filament formation. (**a**) Schematic of the C-AFM measurements on Ti/HfO_2/TiN structure. (**b**) Hysteretic *I–V* characteristics from the Ti/HfO_2/TiN structure during C-AFM measurements, implying bipolar resistance switching behavior. (**c**) Observation of gas bubble formation beneath the top electrode when a positive voltage sweeping was applied. The yellow dot denotes the position where the Pt probe was landed. Scale bar: 2 μm. (**d**) Schematic of the C-AFM measurements on TaO_x/TiN samples. (**e**) *I–V* characteristics when a positive voltage sweeping was performed on the TaO_x/TiN sample. The inset shows structural deformation after resistance switching occurs. (**f**) 3D topographic view of the data shown in the inset of (**e**), which clearly shows the gas eruption and subsequent structural deformation to the TaO_x/TiN structure.

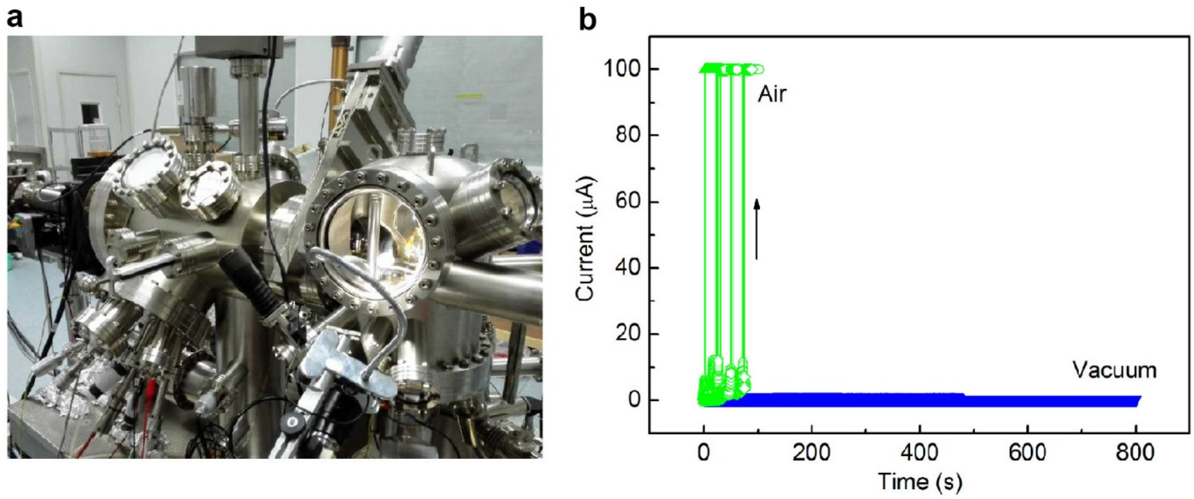

Supplementary Figure 7 | Influence of moisture on resistance switching. (**a**) Experimental setup used for SPM study in the vacuum (~8×10^{-11} mbar). (**b**) Comparison of the switching time in air and in vacuum when the same voltage of 6 V was applied on the HfO_2/TiN sample.

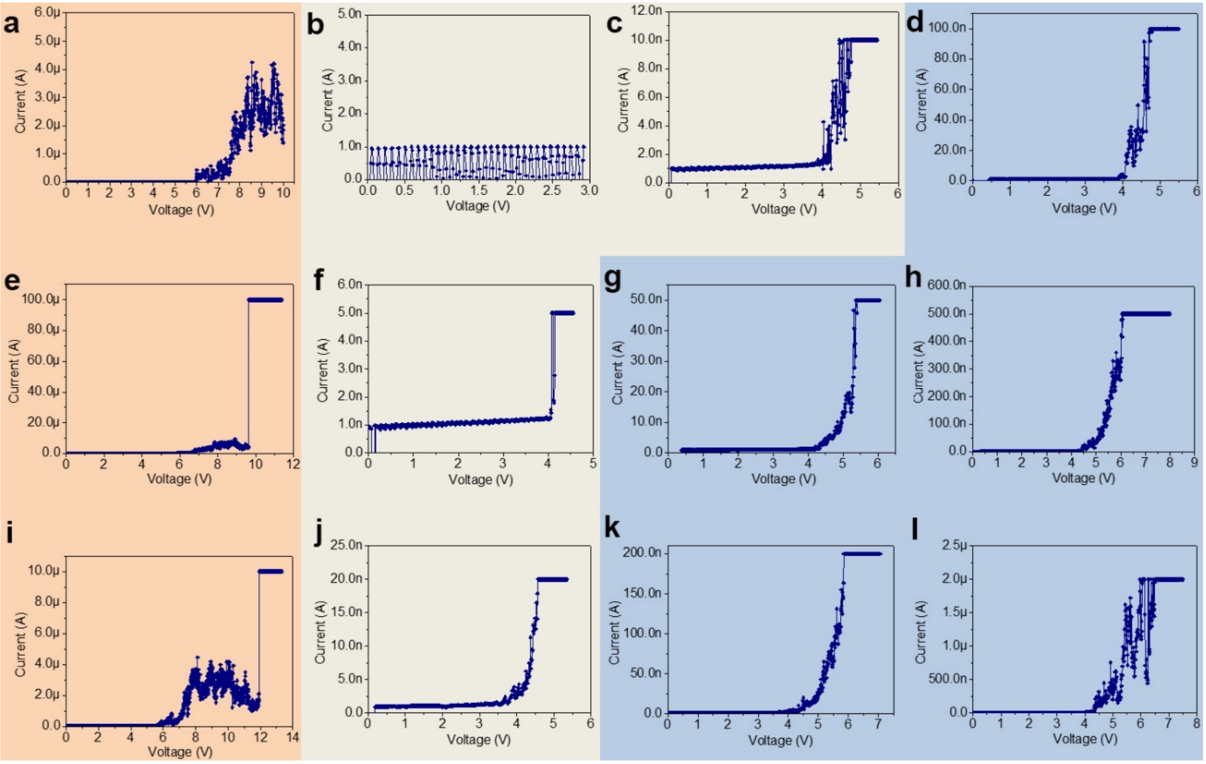

Supplementary Figure 8 | *I–V* characteristics corresponding to the stimulated regions in Fig. 5a. The panels are arranged following the same positions of the simulated regions. The applied current was controlled in three different ranges, namely weak (1–20 nA), moderate (typically 50 nA–2 μA) and strong (4–100 μA) conditions to set the regions into different resistance states. The different colors denote regions where similar programming conditions were applied.

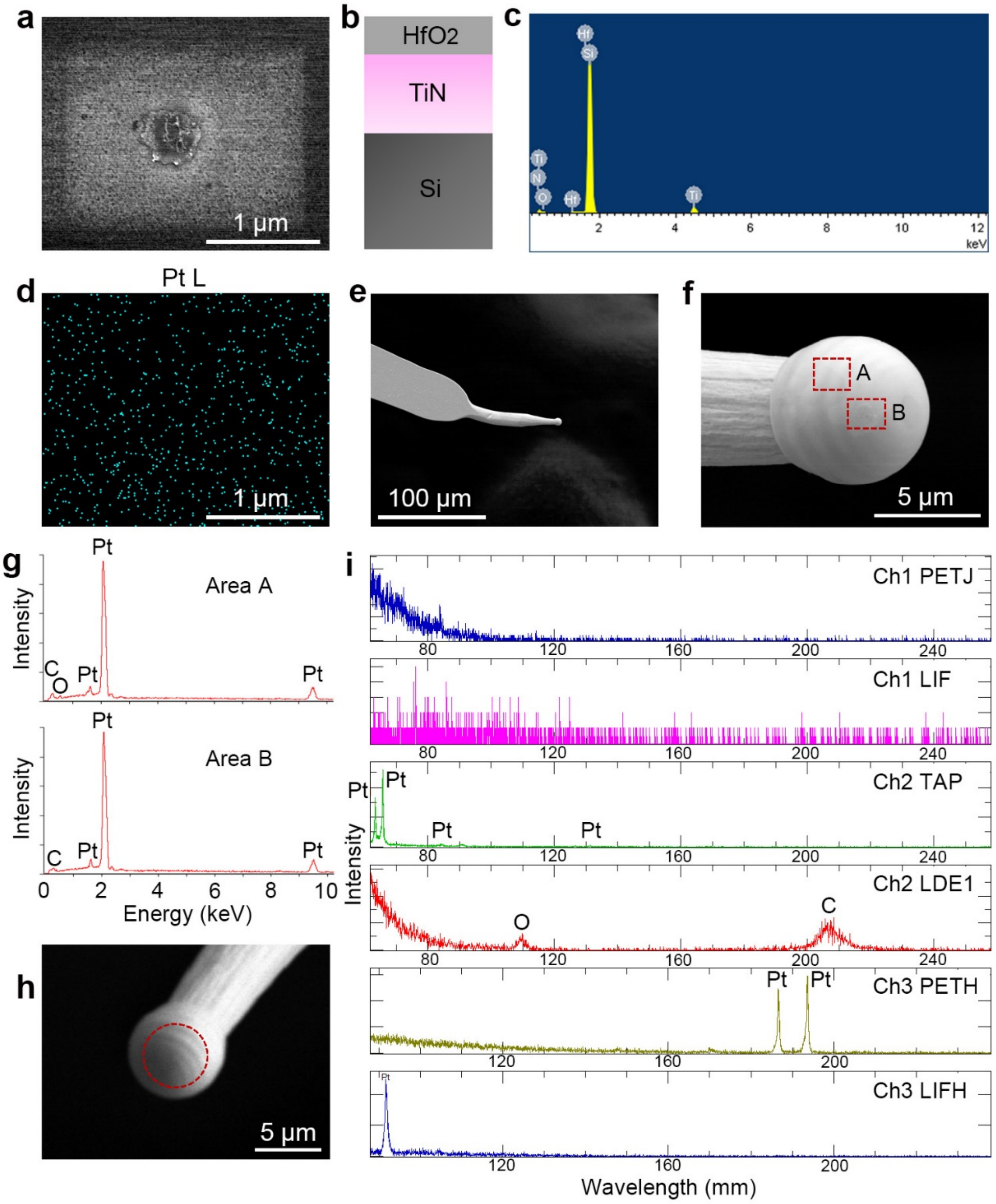

Supplementary Figure 9 | Characterization of tip-sample interactions. (**a**) SEM image of a typical crater that is formed on the HfO_2/TiN sample, whose stacking structure is illustrated in (**b**). (**c**) EDS spectrum from the crater region shown in (**a**), with no signal shown for Pt. (**d**) EDS mapping for the Pt L edge in the same region shown in (**a**). (**e**) SEM image of the Pt tip that was used to create the crater shown in (**a**), and a zoomed-in image is shown in (**f**). (**g**) EDS spectra from the regions “A” and “B” in (**f**) as indicated by rectangles. (**h**) EPMA image of the Pt tip that was used to create the crater, depicting the region for WDS analysis, and (**i**) corresponding WDS spectra from different crystals.

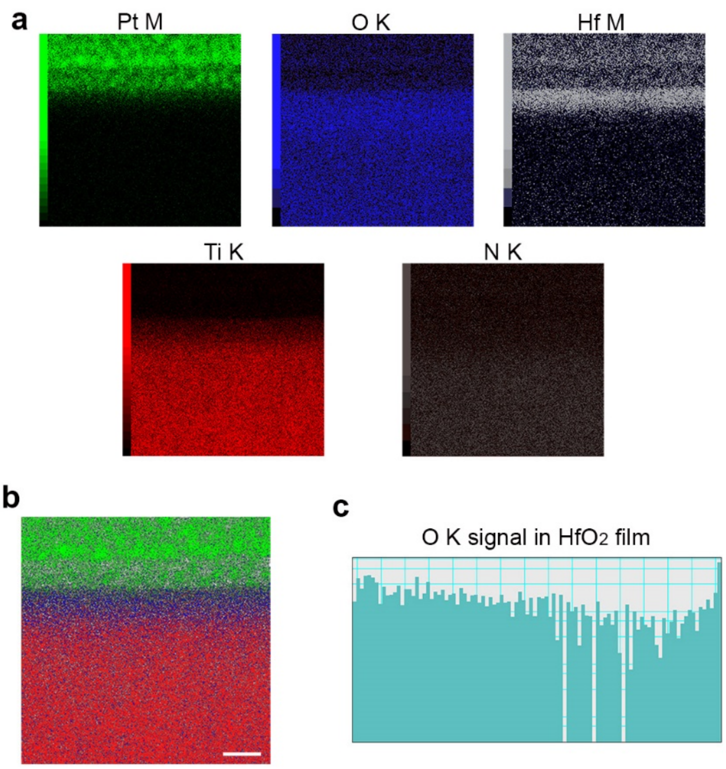

Supplementary Figure 10 | EDS mapping on the pristine state of the HfO_2/TiN sample. (**a**) EDS mapping of Pt M, O K, Hf M, Ti K and N K edges and (**b**) overlaid mapping results in the pristine state of the HfO_2/TiN sample. Scale bar: 20 nm. (**c**) Histogram of O K edge signal intensity in the HfO_2 film in the pristine state. The results suggest highly uniform oxygen distributions.

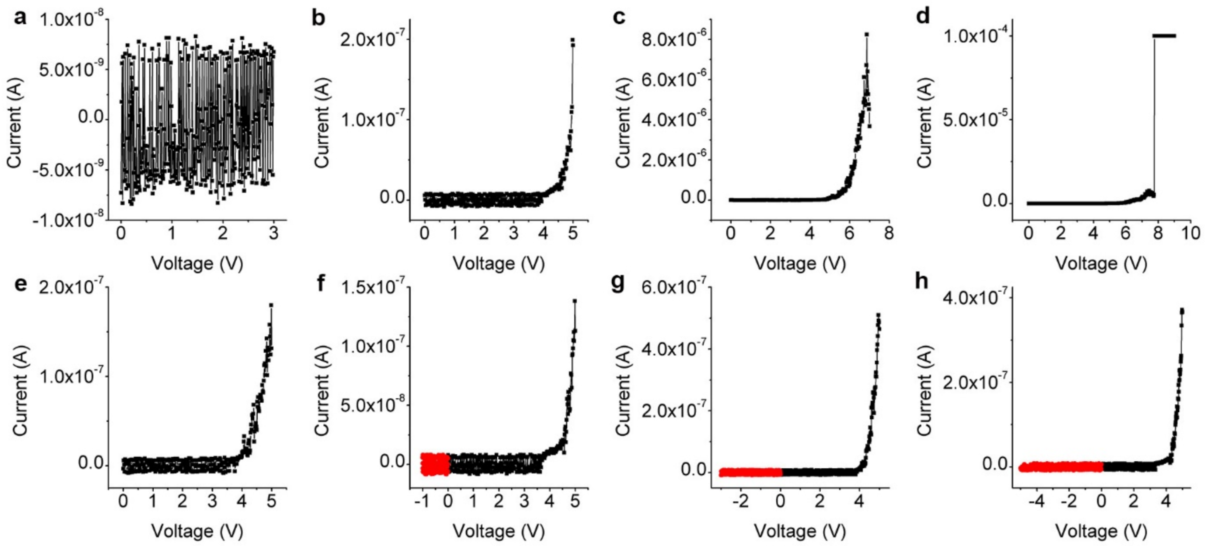

Supplementary Figure 11 | *I–V* characteristics of HfO_2/TiN sample corresponding to the EFM measurements in Figs. 6a–c. The panels are arranged following the same positions of the simulated regions in Fig. 6a for the sake of clarity.

Supplementary Note 1. Nature of the electrostatic force between the probe and the sample

In order to verify the nature of the accumulated charges observed in 1ω signal, we conducted phase detection electrostatic force microscopy measurement (Supplementary Refs. 1–3). The HfO_2/TiN sample was firstly probed by a Pt probe on the surface to perform C-AFM measurements with different DC sweeping voltages, and then phase detection EFM was conducted in lift mode with +2 V tip bias to extract the phase shift. Generally, the cantilever was vibrated by a small piezoelectric element near its resonant frequency in the present EFM measurements. The resonant frequency of the cantilever changes in response to any additional force gradient. In principle, attractive forces make the cantilever effectively "softer", which reduces the resonant frequency, so that the phase shift $\Delta\Phi$ is negative. Conversely, repulsive forces make the cantilever effectively "stiffer", which increases the resonant frequency and shifts phase positively.

With a positive voltage applied to the tip during EFM, as shown in Supplementary Fig. 1, the phase shift $\Delta\Phi$ is more negative in the stimulated region than the rest of the sample, which means the electrostatic force between the tip and the stimulated region is attractive instead of repulsive, further confirming that the charges that locally accumulated near the surface after positive voltage sweeps are indeed negatively charged species, e.g. oxygen ions or trapped electrons.

Supplementary Note 2. EFM characterization after negative voltage sweeps

In order to further verify that the migration and accumulation of oxygen ions can indeed be probed by the 1ω component of the electrostatic force gradient between the probe and the sample, we have further performed comparative EFM studies on HfO_2/TiN samples where negative voltage sweeps are performed in preceding C-AFM measurements (Fig. 3). Supplementary Fig. 2 shows the corresponding current–voltage (*I*–*V*) curves during the negative voltage sweeps. The experiments were performed in two independent runs, and the *I*–*V* curves are shown in Supplementary Figs. 2a–b and Supplementary Figs. 2c–e, respectively. Since the oxygen ions should be driven to the bottom HfO_2/TiN interface due to the reversed electric field direction in the case of negative voltage sweeping, it was confirmed that the previous charge accumulations in Figs. 1f,j,n,r were no longer observed until mechanical distortions took place when the negative voltage exceeded –4 V. The results are highly consistent in the two runs, therefore confirming the correlation between charge accumulation in the 1ω signal and the oxygen ion migration to the interface. This makes EFM a reliable approach to studying oxygen ion motion in oxide memristors.

Supplementary Note 3. EFM characterization using Al_2O_3 as the electrolyte material

In order to verify that the migration of oxygen ions can indeed be probed by the 1ω component of the electrostatic force gradient between the probe and the sample, we have further performed control experiments on Al_2O_3(~5 nm)/TiN samples (Fig. 4). It is well known that Al_2O_3 has extremely low diffusion coefficient of oxygen ions even close to its melting point (~2000 °C). In the meantime, Al_2O_3 has a high activation energy of ~6.5 eV for oxygen diffusion (Supplementary Ref. 4). This decides that the oxygen ion motion in Al_2O_3 should be significantly retarded. Supplementary Fig. 3 shows the *I*–*V* curves of the Pt tip/Al_2O_3/TiN structure in a series of voltage sweeps with different amplitudes (3, 5, 7, 9, 11, 13 V). The samples were then subjected to EFM measurements, which indeed shows the absence of charge accumulation below 11 V (which is a high voltage compared with results in HfO_2). This is also in agreement with the *I*–*V* characteristics in Supplementary Fig. 3, where the current level stays fairly low below 11 V (<50 nA), due to the difficulty of oxygen ion motions in Al_2O_3.

Supplementary Note 4. EFM measurements in vacuum

In order to assess the role of the testing environment especially surface adsorptions, we have performed control experiments in an ultrahigh vacuum SPM setup (Scienta Omicron variable temperature SPM, vacuum level of ~8×10^{-11} mbar, shown in Supplementary Fig. 7a). The sample was also baked at ~140 °C prior to C-AFM and EFM characterization in order to further remove possible adsorbates from the surface, and identical C-AFM and EFM measurements with that in air were performed afterwards. Supplementary Fig. 4 shows the 1ω (see Supplementary Figs. 4b,e,h,k) and 2ω (see Supplementary Figs. 4c,f,i,l) components of the EFM signal between the probe and the switching region after applying voltage sweeps during the preceding C-AFM measurements (Supplementary Figs. 4a,d,g,j). One can see that highly consistent results with that in air (Fig. 1) were obtained, therefore demonstrating the subordinate role of random adsorbates from the ambient in EFM characterization. It should be pointed out that in this new ultrahigh vacuum SPM setup, a metallic multi-walled carbon nanotube with much smaller diameter was used as the probe instead of the conventional Pt-Ir coated silicon tip, which accounts for the smaller area of the charge-accumulation regions (Supplementary Figs. 4b,e,h,k). The 1ω signal is otherwise very similar. In addition, it is well known that the resistance switching process itself (instead of the EFM measurement) will be retarded in vacuum condition, since the moisture in air plays a role in facilitating the redox reactions and charge transfer processes that are indispensable for resistive switching (Supplementary Refs. 5–8). As a result, the switching voltage was increased to ~10 V in vacuum (compared with ~8.4 V in air), and the current level was also much lower due to the current compliance of the equipment (~200 nA, compared with 100 μA in Fig. 1p), as shown

in Supplementary Fig. 4j. The thermal effect during switching was thus significantly reduced, which in turn avoids severe structural damages shown in Fig. 1q and hence prevents pronounced features from being observed in the 2ω signal. All the results are highly consistent with that in ambient conditions and precludes the influence of surface adsorptions.

Supplementary Note 5. Electrostatic force microscopy on lateral devices

In addition to the vertical devices shown in Figs. 1–4 and 6, we have also prepared devices with a lateral configuration using e-beam lithography, as shown in Supplementary Fig. 5a. The device has a nominal electrode distance of ~150 nm. By applying a positive voltage sweep on the right electrode with respect to the left one (see Supplementary Fig. 5b), theoretically oxygen ions are expected to accumulate around the anode. On the contrary, oxygen vacancies originally existing in the as-prepared oxide film should get accumulated at the counter electrode. Supplementary Fig. 5c clearly demonstrates the existences of charge accumulations at the surfaces of both electrodes, as indicated by the dashed lines. However, these may not be directly related to oxygen ions/vacancies, since both the electrodes are present and thus form a capacitor, unlike the previous HfO_2/TiN samples. The charges stored in the capacitor due to preceding voltage applications may complicate the interpretation and assignment of the electrostatic signals. Nevertheless, these results unambiguously demonstrate the proposed technique in the present study indeed has the capability of analyzing lateral thin film devices as well, if the capacitance effect can be safely decoupled.

Supplementary Note 6. Oxygen gas eruption and filament formation in HfO_2 and TaO_x

We have performed further C-AFM measurements on Ti/HfO_2/TiN samples, as schematically shown in Supplementary Fig. 6a. As shown in Supplementary Fig. 6b, *I*–*V* curves from this structure using C-AFM measurements clearly display hysteretic resistance switching that is characteristic for a Ti/HfO_2/TiN memristive structure. Supplementary Figure 6c further shows topographic profile of the Ti/HfO_2/TiN cell after off → on switching, and the yellow dot indicates the region where the probe was landed during the voltage sweep. One can see that a "volcano" shaped bubble is formed at the corner of the Ti electrode, as marked by the circle, which is reminiscent of the structural damages shown in Figs. 1m, 1q and 2a. Such formation of bubbles in oxides based memristors has been well understood to be a result of oxygen ions migration and their subsequent oxidations at the electrode/oxide interface, and the resultant oxygen gas is trapped by the top electrode and thus causes the bubble formation (Supplementary Refs. 9–10). The results in Fig. 1 and Supplementary Fig. 6a–c therefore point to a consistent picture during the resistance switching of HfO_2, where oxygen ions drift as driven by the electric field and the redox reactions at the interface lead to oxygen-deficient filament formation, structural deformation as well as oxygen gas eruption. In the previous measurements on the HfO_2/TiN sample without the top electrode (Figs. 1 and 2), the oxygen gas formed by electrochemical oxidation will directly emit to the ambient and thus was not detected. The introduction of the top electrode in Ti/HfO_2/TiN has therefore played a role in signifying such gas formation.

It is worthwhile pointing out that such oxygen ion motion and accompanying effects are universal phenomena for different oxides. We have performed similar C-AFM experiments on

TaO_x/TiN samples, as shown in Supplementary Fig. 6d, and a sudden resistance switching was observed at ~10 V (Supplementary Fig. 6e). Topographic analysis once again suggests distortions to the TaO_x film as well as formation of oxygen-deficient conduction channels in TaO_x, as shown in the inset of Supplementary Fig. 6e and Supplementary Fig. 6f. These results are fully consistent with that observed in HfO_2 and once again verify that the ion migrations and redox reactions should be responsible for the resistance switching in oxides based memristive devices.

Supplementary Note 7. Effect of moisture on resistance switching

In order to examine the role of ambient condition, e.g. moisture level, in resistance switching of oxides, we have performed additional experiments on the HfO_2/TiN samples using C-AFM in two different environments: i) in air (with moisture, the moisture level was controlled to be ~35%) and ii) in vacuum (with no or limited moisture). The vacuum level of the SPM system (Scienta Omicron VT-SPM) used in the new experiments was $\sim 8\times10^{-11}$ mbar, and the sample was also baked at ~140 °C before measurements in order to further remove the absorbed moisture. The results are shown in Supplementary Fig. 7b. One can see that when the same bias voltage of 6 V was applied on the C-AFM tip, the sample was always switched to on state in less than 100 s in air, as confirmed by >10 repetitive tests. In stark contrast, repetitive measurements have shown that successful switching was not achieved on the same sample in vacuum condition in much longer time, i.e. up to 800 s. These results clearly demonstrate the role of moisture in assisting the redox reactions and charge transfer that are indispensable for successful resistance switching, in agreement with previous reports on the effect of moisture for both VCM and ECM devices (Supplementary Refs. 5–8).

In addition to the above current–time (*I*–*t*) measurements employing fixed voltage biases, *I*–*V* measurements have suggested similar role of moisture in resistance switching. The HfO_2/TiN sample was switched to on state at around ~8.4 V in air (Fig. 1p), however the switching voltage has increased to ~10 V in vacuum, as shown in Supplementary Fig. 4j, once again consistent with the role of moisture in facilitating resistance switching processes.

Supplementary Note 8. Controlled programming of resistance states for systematic STEM and EDS characterization

We have adopted a number of varied switching conditions during C-AFM measurements to locally create a collection of stimulated regions existing at different stages of resistance switching to understand the dynamic switching process as well as the physical mechanism corresponding to different resistance states, as shown in the SEM image in Fig. 5a. Corresponding *I*–*V* characteristics in the programming processes are shown in Supplementary Fig. 8, where the panels are arranged following the same positions of the simulated regions in Fig. 5a. The large number of switching regions increases the probability for including the local conduction channels in the TEM specimen. In Fig. 5a, regions showing obvious structural deformations (as indicated by the arrows) were obtained by applying relatively high programming current, typically in the range of 4–100 μA, as shown in Supplementary Figs. 8a,e,i. These structural deformations are caused by oxygen gas eruption and strong Joule heating after formation of complete filaments during the switching process. Regions existing at pristine and intermediate switching states (as indicated by the solid red and dotted white circles in Fig. 5a, respectively) were obtained by applying relatively weak (1–20 nA) or moderate programming currents (typically 50 nA–2 μA), as shown in Supplementary Figs. 8b,c,f,j and Supplementary Figs. 8d,g,h,k,l, respectively. The different levels of programming conditions are coded by colors in Supplementary Fig. 8. The operation is more similar to the actual device operations under moderate programming conditions, while the pristine states can be used as references for understanding the switching mechanism in HfO_2 based memristive devices.

Supplementary Note 9. Characterization of tip-sample interactions in forming process

In order to figure out if there exists any tip-oxide interaction when the volcanos/craters are created during the electroforming process, we have performed extensive EDS mapping and spectral analysis as well as Electron Probe Microanalyzer (EPMA) characterization and Wavelength Dispersive X-ray Spectroscopy (WDS) analysis. Supplementary Figure 9a shows a typical crater that is formed on the HfO_2/TiN sample, whose stacking sequence in vertical direction is illustrated in Supplementary Fig. 9b. All the elements included in this stacking structure, i.e. Si, Ti, N, Hf and O, were detected in the EDS measurement, however, with no signal indicating existence of Pt (Supplementary Fig. 9c). We have also performed EDS mapping on the Pt L edge, and once again only background noise was collected, as shown in Supplementary Fig. 9d. These results exclude the possibility of alloy formation during the forming process, since the Pt element should get incorporated into the sample in that case, at least in the crater region, which is in contrast to the above experimental results.

In addition to the above characterization on the crater, a complete examination on the alloying possibility should also include analysis on the Pt tip, as shown in Supplementary Figs. 9e–f. It is expected that alloy formation should also lead to cross contamination to the tip. It is interesting to find out that the end of the Pt tip seems to have gone through a melting process and a sphere-like shape was formed, probably due to the accompanying significant Joule heating effects during electroforming. In order to further check on the possibility of alloy formation, two areas labeled as "A" and "B" were subjected to EDS analysis. While region A is the pristine Pt tip, region B was in direct contact with the HfO_2 surface during the C-AFM measurements, which has been identified by the existence of a small flat surface in region B

due to the tip-sample contact. Supplementary Fig. 9g shows that both regions are very similar in composition and only Pt can be detected (besides C and O that are well-known contamination elements from the ambient), while characteristic peaks from Hf or Ti cannot be observed, once again ruling out the possibility of alloy formation.

These conclusions were further testified by WDS analysis in EPMA, whose sensitivity in compositional analysis is about one order of magnitude higher than that of EDS, however at the cost of spatial resolution (Supplementary Ref. 11). Supplementary Fig. 9h shows the analyzed region by EPMA-WDS, where region B in Supplementary Fig. 9f has been included in the analyzed region, and the results from different types of crystals and channels once again exclude the existence of Hf or Ti on the Pt probe (Supplementary Fig. 9i). Unfortunately, such analysis could not be conducted on the nanoscale craters (~500 nm in diameter), due to the spatial resolution limitation of EPMA as mentioned above.

We believe based on the extensive analysis shown above, it can be safely concluded that due to the extreme programming conditions during the forming process, *physical* interactions occur between the Pt tip and the HfO_2/TiN sample, mainly manifesting as Joule heating effects as suggested by the melted end of the Pt tip, which is also consistent with our TEM observations as discussed in the main text. However, *chemical* interactions between the probe and the sample, such as alloy formation, seem to be absent.

Supplementary Note 10. STEM and EDS characterization on pristine HfO_2

Supplementary Fig. 10a shows EDS mapping of Pt M, O K, Hf M, Ti K and N K edges from the pristine state of the HfO_2/TiN sample, and Supplementary Fig. 10b further shows the overlaid mapping results. Supplementary Fig. 10c further exhibits the histogram of O K edge signal intensity in the HfO_2 film. One can see that in the pristine state of the sample, the concentration distribution of the oxygen element is highly uniform. This is in contrast to the non-uniform distribution of oxygen concentration in the locally switched HfO_2 region, as shown in Figs. 5h–o, hence suggesting formation of a conducting filament in the latter case.

Supplementary Note 11. Reversible ion motion in forming and reset processes of HfO_2 memristors

The mechanism of reset process in HfO_2 memristors was also studied by C-AFM and EFM characterizations, as shown in Fig. 6, and Supplementary Fig. 11 shows the corresponding *I–V* characteristics. The panels are arranged following the same positions of the simulated regions in Fig. 6a. When a positive voltage of 5 V was applied, Supplementary Figure 11b shows that a relatively high current level of ~200 nA has been reached, therefore indicating the onset of conducting filament formation under 5 V. Figures 6a and 6c also show that when the applied voltage further increased structural distortions will take place. As a result, we have adopted 5 V as the forming threshold. In the second run of experiments (bottom part of Figs. 6a–c), the HfO_2/TiN sample was firstly stimulated by a positive voltage sweep to 5 V, which attracts oxygen ions to the top interface, followed by negative voltage sweeps in the same locations with gradually increased negative voltages (0, –1, –3, and –5 V). Backward ion migration was clearly observed in Fig. 6b, where the oxygen ions accumulated at the top interface gradually disappeared. This illustrates the reversible ion motion in forming and reset processes, accounting for the bipolar resistance switching process in HfO_2 memristors.

Supplementary References

1. Burnett, T., Yakimova, R. & Kazakova, O. Mapping of local electrical properties in epitaxial graphene using electrostatic force microscopy. *Nano lett.* **11**, 2324-2328 (2011).
2. Lei, C. H. *et al.* Quantitative electrostatic force microscopy-phase measurements. *Nanotechnol.* **15**, 627-634 (2004).
3. Girard, P. Electrostatic force microscopy: principles and some applications to semiconductors. *Nanotechnol.* **12**, 485-490 (2001).
4. Heuer, A. H. Oxygen and aluminum diffusion in α-Al_2O_3: How much do we really understand? *Journal of the European Ceramic Society* **28**, 1495-1507 (2008).
5. Lübben, M., Karakolis, P., Ioannou-Sougleridis, V., Normand, P., Dimitrakis, P. & Valov, I. Graphene-modified interface controls transition from VCM to ECM switching modes in Ta/TaO_x based memristive devices. *Adv. Mater.* **27**, 6202–6207 (2015).
6. Messerschmitt, F., Kubicek, M. & Rupp, J. L. M. How does moisture affect the physical property of memristance for anionic-electronic resistive switching memories? *Adv. Funct. Mater.* **25**, 5117–5125 (2015).
7. Tappertzhofen, S., Valov, I., Tsuruoka, T., Hasegawa, T., Waser, R. & Aono, M. Generic relevance of counter charges for cation-based nanoscale resistive switching memories. *ACS Nano* **7**, 6396–6402 (2013).
8. Tsuruoka, T., Terabe, K., Hasegawa, T., Valov, I., Waser, R. & Aono, M. Effects of moisture on the switching characteristics of oxide-based, gapless-type atomic switches. *Adv. Funct. Mater.* **22**, 70–77 (2012).

9. Kwon, D. H. *et al.* Atomic structure of conducting nanofilaments in TiO_2 resistive switching memory. *Nat. Nanotechnol.* **5,** 148-153 (2010).

10. Yang, J. J. *et al.* The mechanism of electroforming of metal oxide memristive switches. *Nanotechnol.* **21,** 215201 (2009).

11. Laigo, J., Christien, F., Le Gall, R., Tancret, F. & Furtado, J. SEM, EDS, EPMA-WDS and EBSD characterization of carbides in HP type heat resistant alloys. *Mater. Charact.* **28**, 265-291 (2004).
